# Long-Term Patient-Reported Quality-of-Life Outcomes After Ventral Primary or Incisional Hernia Repair

**DOI:** 10.3390/jcm15207970

**Published:** 2026-10-15

**Authors:** Tara K. He, Joanna M. Z. Mills, Nazim Bhimani, Anthony R. Glover, Thomas J. Hugh

**Affiliations:** 1 Northern Clinical School, Sydney Medical School, The University of Sydney, Sydney, NSW 2006, Australiajoannamannzhuang.mills@anu.edu.au (J.M.Z.M.); 2 Sydney Medical School, The University of Sydney, Sydney, NSW 2006, Australia; nazim.bhimani@health.nsw.gov.au (N.B.); anthony.glover@sydney.edu.au (A.R.G.); 3 Upper Gastrointestinal Surgical Unit, Royal North Shore Hospital, Sydney, NSW 2065, Australia

**Keywords:** ventral hernia, incisional hernia, quality of life, patient-reported outcomes

## Abstract

**Background**: Long-term quality-of-life (QOL) outcomes after ventral primary and incisional hernia repair are underreported, especially beyond the early postoperative period. This cross-sectional study evaluated postoperative functional status, mental health and overall satisfaction using patient-reported outcome measures (PROMs) at least five years after ventral hernia surgery. **Methods**: Four separate PROM assessment tools were used to assess long-term outcomes in patients who underwent ventral hernia repair between 2009 and 2014. Two subgroup analyses were performed, categorizing patients by recalled preoperative pain and Ventral Hernia Working Group risk classification (low-risk Grade 1/2 vs. high-risk Grade 3/4), based on wound contamination and comorbidities at the time of surgery. **Results**: Eighty-seven patients were assessed with a median follow-up of eight years (range 6–11) from the time of surgery to survey completion. Patients with Grade 3/4 hernias (18%, 16/87) reported significantly worse QOL outcomes across multiple PROMs than those in the Grade 1/2 group (82%, 71/87), including social functioning (*p* = 0.010), pain (*p* = 0.013), general health (*p* = 0.007), and cosmetic discomfort (*p* = 0.018). Patients with moderate to severe preoperative pain (53%, 46/87) were more likely to experience long-term postoperative pain (*p* = 0.02) and worse social functioning (*p* = 0.004) compared with patients with minimal or no preoperative pain (47%, 41/87). **Conclusions**: Overall, many patients had favorable long-term patient-reported QOL outcomes following ventral primary and incisional hernia repair. Patients with high-risk, complex hernias and those with moderate-to-severe recalled preoperative pain experienced more pain and worse overall health at long-term follow-up than those with low-risk hernias and those with minimal or no recalled preoperative pain.

## 1. Introduction

Ventral primary and incisional hernia repair are common elective procedures, yet few studies explore long-term patient-reported quality-of-life (QOL) outcomes such as pain, functional impairment and psychosocial wellbeing. Short-term QOL studies have shown that preoperative pain, diabetes, smoking, obesity, and large hernia size are associated with higher rates of complications, including pain and surgical site infections (SSIs) [1,2]. These outcomes may impact on mental health, daily function, and perceptions of surgical success [1,2]. In contrast, long-term prospective longitudinal studies exploring the same QOL outcomes are rare. Most outcomes focus on either short-term follow-up (weeks up to a maximum of five years), or on non-QOL outcomes including postoperative complications, mortality, and recurrence [3,4].

The population-adjusted incidence of hernias is rising in Australia, with abdominal wall repairs increasing from 38,223 to 49,624 over the past decade [5]. In conjunction with developments in operative techniques and complexity, this trend has been accompanied by a shift in the definition of hernia repair success toward QOL and patient satisfaction, particularly in patients who undergo low-risk, elective operations [1,6,7]. Despite this, <1% of hernia studies assess long-term QOL using validated patient-reported outcome measures (PROMs), which are self-reported, standardized questionnaires that quantify symptoms, function, and QOL to assess surgical outcomes and support patient-centered care [4,8].

Recently, our group published long-term clinical outcomes after primary ventral and small (≤2 cm) incisional hernia repair over a median follow-up period of 12 years, focusing on operative techniques (primary suture repair vs. sandwich mesh repair) and patient-reported outcomes, including SSI, pain, discomfort and recurrence [9]. The present study expands this work by including all ventral hernia types irrespective of size or complexity and focuses on long-term QOL, psychosocial and functional outcomes measured using four separate validated PROMs: the 36-Item Short Form Survey (SF-36) [10], the Ventral Hernia Pain Questionnaire (VHPQ) [11], the European Hernia Society Quality of Life (EuraHS-QOL) [12], and the Carolinas Comfort Scale (CCS) [13]. Although prospective studies incorporating preoperative and postoperative PROMs provide the strongest assessment of QOL changes, such long-term data are rare. Often, outcome studies are limited to cross-sectional evaluation without prospectively collected baseline PROMs, preventing direct quantification of changes in QOL over time. Despite this limitation, these studies still provide valuable information regarding the efficacy of hernia repair. The aim of the current cross-sectional study is to assess long-term QOL following ventral hernia repair using PROMs collected from patients at least five years after their operation. A secondary aim was to explore whether preoperative pain and hernia complexity were associated with postoperative QOL and chronic pain.

## 2. Materials and Methods

### 2.1. Study Design

Patients who underwent ventral hernia repair between January 2009 and December 2014 under the care of the senior author (TJH) were identified through a retrospective review of prospectively collected clinical data. Eligible patients were contacted as part of the follow-up process between July and August 2020 through invitation letters containing contact information, a QR code, and a survey link. The questionnaire was available in paper format with a prepaid return envelope, or via a personalized link to an online survey through REDCap (version 17.3.10), a secure software platform provided by the Northern Sydney Local Health District for managing surveys and electronic data [14]. Non-responders received up to two follow-up phone calls at four-week intervals. Informed consent was obtained from all participants, and participation was voluntary with the option to withdraw at any point. The consent form and questionnaire can be found in the Supplementary Materials (Figures S3 and S4).

Relevant clinical data were manually extracted from the database and categorized into variables including demographics, operation duration, hernia type (etiology, site, recurrence), admission length (day-only/≥1 night), anesthesia type (local/general), surgical technique (open/laparoscopic), mesh usage (yes/no), primary ventral/incisional status, first-time/multiple recurrence, and mesh type (polypropylene, Gore-Tex Dual Mesh, other).

Two subgroup analyses were performed. Patients were stratified into Grade 1/2 (low-risk) versus Grade 3/4 (high-risk) based on the Ventral Hernia Working Group (VHWG) classification [15]. Grade 1 hernias have clean wounds in otherwise healthy patients, and Grade 2 includes patients with comorbidities such as diabetes, COPD, immunosuppression, active smoking and clinical obesity [15]. Grade 3 patients include potentially contaminated wounds with risk factors such as previous wound infection, a stoma present and a violation of the gastrointestinal tract, while Grade 4 hernias include high-risk patients with infected mesh or septic dehiscence [15]. At long-term follow-up, patients were also classified by the presence or absence of retrospectively recalled preoperative abdominal pain using question one of the VHPQ on a 6-point Likert scale (0 = no pain, 6 = severe pain). Patients reporting no pain or mild, ignorable pain (scores of 0–1) were assigned to a no-preoperative-pain group, while those with moderate to severe, unignorable pain (scores of 2–6) were assigned to a preoperative-pain group. No additional analyses were performed to explore the relationship between hernia VHWG grade and recalled preoperative pain status. Follow-up duration was calculated from the date of surgery to the date of survey completion.

### 2.2. Operative Approach

All operations were done as either an elective or semi-urgent procedure and were performed or supervised by the senior author (TJH). Patients received a single dose of prophylactic intravenous cephalosporin at induction. An aqueous-based skin preparation was applied routinely, followed by preemptive skin and subcutaneous tissue infiltration with 4–5 mL of 0.75% Ropivacaine (AstraZeneca, Cambridge, UK). Thromboprophylaxis included mechanical on-table calf stimulators for day-only patients and subcutaneous heparin 5000 units twice daily for inpatients.

Both open and laparoscopic repairs were included in this study. Laparoscopic repairs were performed exclusively for Grade 1 and 2 hernias while Grade 3 and 4 hernias were preferentially managed with open repair because of the greater complexity and the requirement to remove previously placed mesh in many patients. As the surgical approach was inherently determined by the hernia grade and complexity, no comparison of outcomes between open and laparoscopic repairs was undertaken.

The three main types of open operations included primary suture closure repair for hernias ≤ 10 mm, primary sutured closure with or without component separation for contaminated wounds, or extraperitoneal partial tension sandwich repair using polypropylene mesh (Prolene mesh, Ethicon, North Ryde, Australia) for clean or clean-contaminated hernias using a technique described previously [9].

All laparoscopic operations were completed as an intraperitoneal onlay mesh (IPOM) repair using ePTFE (Gore-Tex Dual Mesh, W. L. Gore & Associates, Inc., Newark, DE, USA) as described previously [16]. Mesh or suture material was removed as required at the time of the hernia repair, and selected patients underwent bowel resection in the presence of sinus tracts or fistulae. Patients underwent routine follow-up at 4–6 weeks postoperatively, and continuing for two years or more depending on individual complications and comorbidities.

In this study, surgical site infection (SSI) was broadly defined as the presence of ‘erythema’ in or around the wound or a positive culture of an organism from a wound swab. Given the frequent use of mesh, all patients with a suspected SSI were treated early and proactively with oral antibiotics.

### 2.3. Survey Questionnaire

At long-term follow-up, patients completed one generic and three hernia-specific PROMs. The SF-36 is the most commonly used generic PROM in hernia repair studies and assesses eight domains—physical functioning, role limitations due to physical health, role limitations due to emotional health, energy/fatigue, emotional well-being, social functioning, bodily pain, and general health perceptions—with higher scores indicating better self-reported QOL [10,17]. The hernia-specific PROMs used in this study are also commonly used in hernia research [17] and include the VHPQ [11], the EuraHS-QOL [12], and the CCS [13]. The VHPQ consists of 20 questions assessing pain duration and severity, impact on daily activities, and patient satisfaction using both a numerical seven-step fixed-point rating scale and categorical responses [11]. The EuraHS-QOL instrument comprises nine questions across three domains—pain, activity restrictions, and cosmetic discomfort—with total scores ranging from 0 (better QOL) to 90 (worse QOL) [12]. The CCS is a 23-item Likert-type questionnaire scored from 0 (better QOL) to 115 (worse QOL) that focuses on mesh sensation and measures pain severity, sensation, and movement limitations [18].

### 2.4. Statistical Analysis

Categorical variables were presented as counts and percentages and analyzed using the Chi-squared test. Continuous variables were expressed as means and standard deviations and analyzed using the independent Student T-test, where the data were normally distributed. In cases of non-normal distribution, median and range were reported, and variables were analyzed using the Mann–Whitney U test. The data was analyzed by a biostatistician (NB) using Stata^®^ BE for Windows^®^ version 17.1 (StataCorp, College Station, TX, USA). Subgroup analyses were unadjusted for potential confounding variables and were therefore considered exploratory.

## 3. Results

### 3.1. Respondent Characteristics

**Figure 1:**
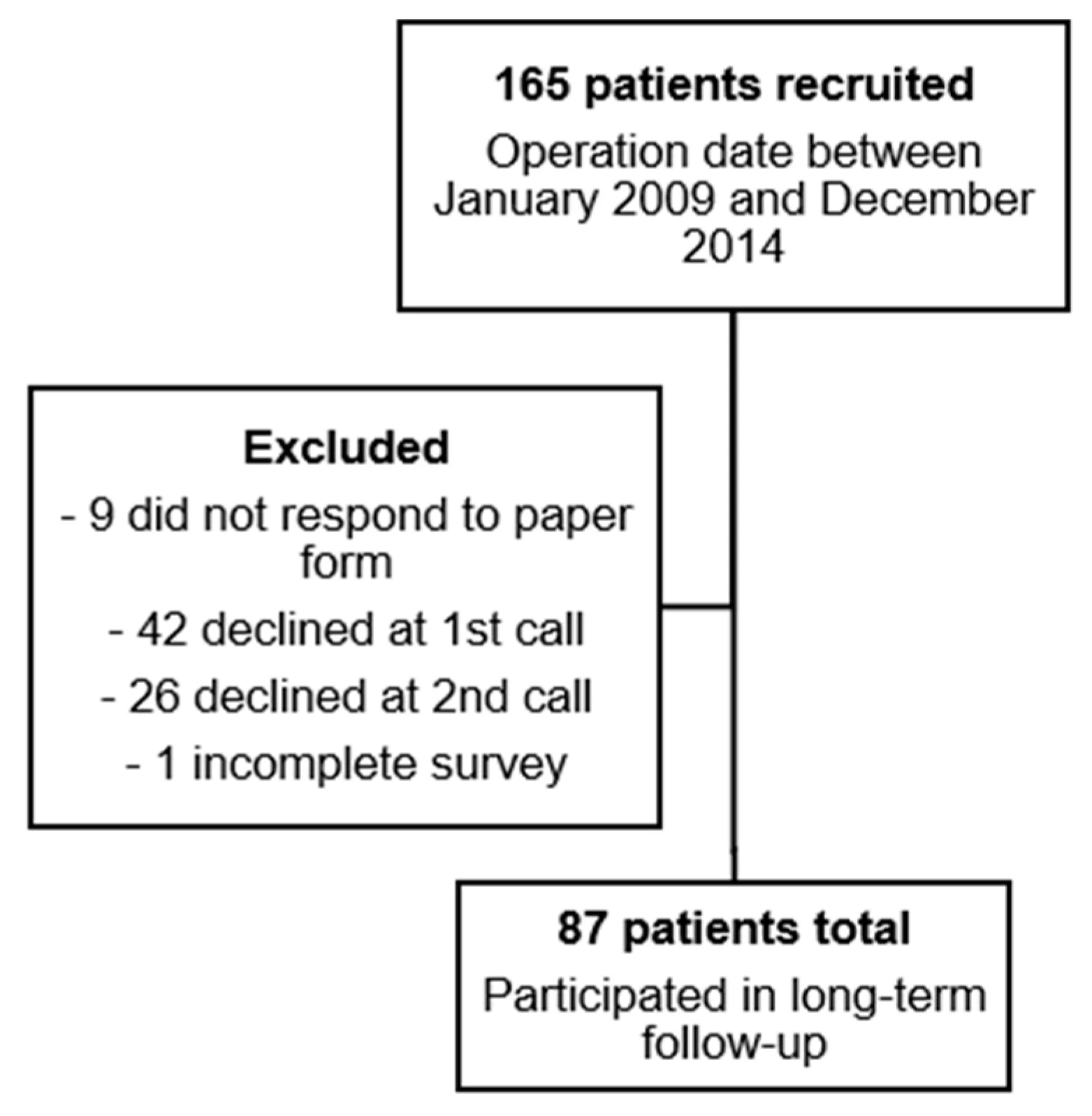
Flow diagram of patients identified in the clinical database for inclusion in the follow-up study and reasons for exclusion over 6–11 years.

From a prospectively maintained clinical database, 165 patients who underwent ventral primary or incisional hernia repair between January 2009 and December 2014 were contacted and 87 patients responded, resulting in a total response rate of 53% (87/165) (Figure 1). One patient did not complete the VHPQ component of the survey and was excluded. Two patients who did not complete, or only partially completed, the SF-36 were included in the overall analysis but were excluded from the subgroup analysis of the SF-36. There were minimal demographic differences between responders and non-responders, with comparable gender distribution (57% vs. 53% male), median age (74 vs. 76 years), and median age at operation (62 vs. 64 years).

### 3.2. Patient Demographic and Surgical Characteristics

A total of 87 patients completed the VHPQ and were included in the final analysis. Patients completed the survey a median of eight years postoperatively, with a range of 6–11 years. The majority of patients were classified as having Grade 1/2 hernias (82%, 71/87) compared to patients with Grade 3/4 hernias (18%, 16/87). In the recalled pain subgroup analysis, 53% (46/87) reported moderate to severe preoperative abdominal pain, and 47% (41/87) reported no preoperative pain. Compared to Grade 1/2 patients, Grade 3/4 patients were significantly older at the time of their operation (median 68 vs. 61, *p* = 0.009), included more women (69% vs. 37%, *p* = 0.019), and had a higher prevalence of incisional hernias (88% vs. 30%, *p* < 0.001). There was a trend toward higher overall postoperative complications (25% vs. 13%, *p* = 0.247), longer operative times (median 70 vs. 40 min, *p* < 0.001), and a much lower proportion of day-only procedures (12% vs. 68%, *p* < 0.001) in the Grade 3/4 group than in the Grade 1/2 group. Patients with incisional hernias were more likely to have moderate-to-severe recalled preoperative pain than those with primary ventral hernias (*p* = 0.049) (Table S1). Patients with incisional hernias were also older at the time of surgery (median 66 vs. 60 years), had longer operative times (median 70 vs. 35 min), and were more likely to have hernias ≥ 2 cm in size (74% vs. 31%) compared with patients with primary ventral hernias.

Small hernias (<2 cm) were present in 52% (45/87) of patients, while hernias measuring ≥2 cm accounted for 48% (42/87) of the cohort. Patients with Grade 3/4 hernias were significantly more likely to have hernia diameters of 2 cm or larger compared to those with Grade 1/2 hernias (75% vs. 42%, *p* = 0.026). Similarly, postoperative pain was more common in patients with hernias ≥2 cm than in patients who had no or insignificant recalled preoperative pain (63% vs. 32%, *p* = 0.004). Thirteen patients (15%) experienced postoperative complications, with superficial wound infection (as defined in the Methods Section) requiring antibiotic treatment being the most frequent complication. All operations were performed under general anesthesia, except for one patient with a small primary ventral hernia who underwent an operation under local anesthetic. The surgical technique was classified as either an open procedure (86%, 75/87) or a laparoscopic IPOM (14%, 12/87). Most open procedures were completed using a partial tension, extra-peritoneal polypropylene sandwich mesh repair technique (80%, 70/87), and only a small proportion of patients (6%, 5/87) underwent a primary sutured repair. Polypropylene mesh was the most commonly used mesh (82%, 71/87), followed by Gore-Tex mesh (11%, 10/87). Six patients (7%, 6/87) had other types of repairs, including five patients who had non-mesh primary suture repairs and one patient who had a complex sandwich repair using Surgisis (biological mesh) as the inner layer and Proceed mesh (polypropylene, Ethicon North Ryde, Australia) as the outer layer (Table S1).

### 3.3. 36-Item Short-Form Survey

A total of 85 patients fully completed the SF-36 and were included in the subgroup analysis. Only one patient partially completed the survey. At long-term follow-up, Grade 3/4 patients reported significantly worse social functioning (*p* = 0.010), pain (*p* = 0.013), and general health (*p* = 0.007), consistent with the greater surgical complexity associated with higher-risk hernia patients. However, physical function was significantly worse in the Grade 1/2 cohort compared to Grade 3/4 (*p* = 0.003). Patients with no recalled preoperative pain generally reported higher SF-36 scores and better QOL at long-term follow-up. Moderate-to-severe pain was associated with significantly lower social functioning (*p* = 0.004) (Table 1).

**Figure 2:**
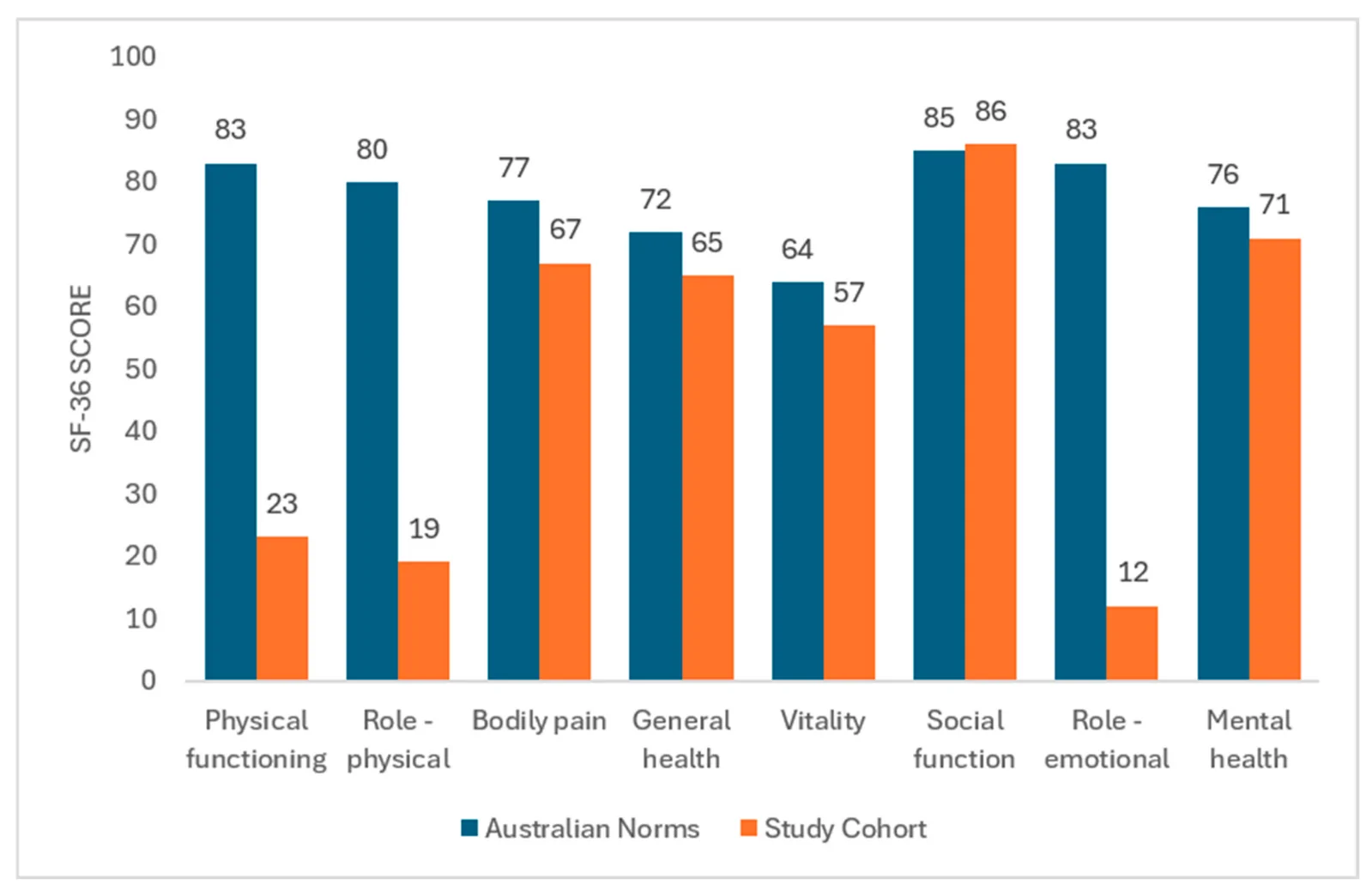
Descriptive contextual comparison of the mean SF-36 scores between patients with ventral hernias (*n* = 85) and 1995 Australian norms.

When SF-36 mean scores of ventral hernia patients in the current study were compared with the 1995 National Health Survey Australian population norms [19], the study cohort demonstrated lower scores in terms of physical functioning (23 vs. 83), physical role limitations (19 vs. 80), and role limitations due to emotional problems (12 vs. 83) (Figure 2).

### 3.4. Ventral Hernia Pain Questionnaire

At the time of survey completion, 10% (9/87) of patients reported currently experiencing moderate to severe pain that could not be ignored (Figure S1). The nature of the pain, whether it was constant, intermittent, or related to specific triggers, was not specified. Additionally, 7% (6/87) of the participants occasionally used painkillers for their pain. Among the 90% (78/87) of patients who reported no pain or ignorable pain at the time of follow-up, the majority (70%, 61/87) indicated that the pain they experienced in the post-operative period had resolved within three months of the operation (Table S2).

Most patients did not experience difficulties with functional activities such as driving, exercise, or movement after surgery. Additionally, the majority (59%, 51/87) reported that their long-term ability to work was not affected by the prior hernia operation or any abdominal pain. Among the employed patients, 6% (2/34) of patients required one to seven days of sick leave in the last two months due to abdominal pain. At follow-up, most patients had not undergone further abdominal surgery, most expressed satisfaction with the procedure, and most indicated that they would undergo an operation again if necessary. Eleven patients reported requiring further unrelated abdominal surgery, including two patients who had a prostatectomy, two who had a separate groin hernia operation, and two who had an unspecified but unrelated abdominal procedure. Four patients experienced hernia recurrence and were subsequently treated by the senior author (TJH). One patient developed a new ventral or incisional hernia at a different site from the original hernia.

### 3.5. European Hernia Society-Quality of Life Instrument

The EuraHS-QOL instrument showed that patients reported minimal postoperative pain, little to no activity limitations, and generally neutral perceptions of cosmetic outcomes. Most patients did not experience pain at the hernia repair site while resting (92%, 80/87) or during activities (90%, 78/87). Daily activities inside and outside the home (92%, 80/87) were largely unrestricted. Among the patients that engaged in sports or heavy labor, 94% (73/78) and 80% (60/75), respectively, reported no activity limitations. Approximately half of the respondents rated the appearance of their abdomen as “beautiful” or “very beautiful” (49%, 43/87), with similar proportions expressing the same sentiment about their hernia site and scar (52%, 45/87). Only 13% (11/87) rated the hernia site and scar as “ugly.”

The EuraHS-QOL total score was significantly lower in the high-risk Grade 3/4 group (*p* = 0.030), with a median score of 11 compared to 4 in Grade 1/2 group, indicating better QOL in the lower-risk group. Additionally, the cosmetic discomfort domain was worse in Grade 3/4 patients (10 vs. 3, *p* = 0.018). Hernia grade did not significantly affect postoperative pain levels or activity restrictions (Table 2).

Recalled preoperative pain levels were significantly associated with postoperative pain scores, with patients who experienced moderate to severe preoperative pain reporting higher postoperative pain scores than those with no preoperative pain (*p* = 0.019). However, there were no significant differences between the groups in terms of activity limitations. Preoperative pain levels also did not significantly influence patients’ postoperative perceptions of the cosmetic appearance of their abdomen or scar (Table 2).

### 3.6. Carolinas Comfort Scale

The CCS results indicated that most respondents experienced minimal long-term postoperative discomfort, with 66% (57/87) reporting no symptoms (CCS total score of 0). Additionally, none of the participants reported severe or disabling symptoms in any category. The CCS scores showed no significant differences between groups, suggesting that sensation, pain, and movement limitations were relatively consistent even in cases of more complex Grade 3/4. There were no statistically significant differences in mesh sensation, pain, or movement between the group with and without pre-operative pain (Table 3).

## 4. Discussion

This study presents long-term follow-up (median eight years, range 6–11 years) reporting on QOL after primary ventral or incisional hernia repair. Most patients reported satisfaction with their operation, maintaining functional independence in daily activities, and experiencing minimal work limitations due to abdominal pain.

### 4.1. Hernia Grade and Associated QOL Outcomes

The VHWG classification serves as a clinically useful tool for risk stratification, and this study affirms the prognostic utility of this system in relation to long-term QOL outcomes. High-risk (Grade 3/4) hernias, reflecting more complex and comorbid patients, were associated with worse QOL across multiple domains with the exception of the physical functioning domain. Compared with the low-risk group, these patients were older, were more likely to have incisional and larger hernias, and underwent longer operations with fewer day-only procedures, consistent with a frailer physiological state and a more complex surgical profile.

Patients with Grade 3/4 hernias reported worse general health, social functioning, and pain scores on the SF-36 as well as higher total EuraHS-QOL scores, suggesting greater functional limitations and symptom burden than patients with Grade 1/2 hernias. However, patients with Grade 3/4 hernias also reported better physical functioning than those with Grade 1/2 hernias. Although statistically significant (*p* = 0.003), this finding should be interpreted with caution given the disparity in group size between patients with Grade 3/4 hernias (*n* = 16) and Grade 1/2 hernias (*n* = 71), raising the possibility of a type I error. Other potential explanations include confounding by non-hernia comorbidities, given that the SF-36 is the only non-hernia-specific PROM used. Survivorship bias is also possible, whereby survivors of infected abdominal complications within the Grade 3/4 cohort may have been a relatively healthier subset.

In contrast, CCS scores did not differ significantly between groups, suggesting that long-term physical symptoms such as chronic pain may not be determined solely by surgical complexity. Worse cosmetic domain scores in the Grade 3/4 group highlight the relevance of appearance-related outcomes following more extensive procedures. Given the subjective nature of cosmetic satisfaction, further research could explore associations between patient factors, incision characteristics, and the perception of outcomes.

Although the number of postoperative complications was higher in the high-risk group compared to the low-risk group, the difference did not reach statistical significance, likely due to the modest sample size. The relatively small number of patients in the Grade 3/4 group (*n* = 16) limited the statistical power of the analysis.

### 4.2. Recalled Preoperative Pain Influencing Postoperative Outcomes

Recalled preoperative pain was associated with poorer long-term outcomes, a finding consistent with previous studies [1]. Patients with moderate to severe preoperative pain reported worse social functioning on the SF-36 and higher postoperative pain levels on the EuraHS-QOL than those with minimal or no preoperative pain. These findings align with broader evidence suggesting that chronic pain affects multiple QOL domains [20,21], and may have contributed to the below-average SF-36 scores observed in this study group compared with Australian population norms. Although chronic pain is known to impair QOL, the extent to which it contributes to reduced outcomes in Grade 3/4 hernias compared with surgical complexity is unclear. Multivariable analyses controlling for pain levels may help clarify this relationship in future studies. Overall, preoperative pain may be relevant to preoperative counselling and setting realistic expectations regarding ongoing postoperative pain, whilst PROMs can be used concurrently to monitor functional and QOL outcomes.

### 4.3. Additional Insights from the PROMs

PROMs can be time-consuming to administer and analyze, and the clinical usefulness is partly limited by the absence of consensus on the most appropriate PROM and timing. Nevertheless, their value in capturing patient-centered outcomes, especially over the long term, justifies continued efforts to standardize their use [17,22,23,24].

In the current study, the majority of patients reported favorable long-term outcomes across all three hernia-specific PROMs—the VHPQ, EuraHS-QOL, and CCS. The cohort was largely composed of patients undergoing open sandwich mesh repair (80%), with minimal chronic pain and functional limitations and high patient satisfaction. Eighty-nine percent of patients reported they were pleased with the operation and 80% stated they would undergo the procedure again. These outcomes suggest that, when appropriately applied, open sandwich mesh repair can achieve durable long-term results in both symptom control and patient satisfaction. However, owing to the lack of a comparator group and small sample sizes in the suture and laparoscopic repair subgroups, conclusions about the superiority of this approach cannot be made. Additionally, because the recurrence data were limited to a few documented cases and the follow-up data did not include all patients undergoing these procedures, it is not possible to state a meaningful recurrence rate.

Despite patients reporting favorable hernia-specific outcomes, comparison of long-term SF-36 scores with Australian population norms highlighted sustained deficits in physical function, role limitations, and emotional domains. Future research would benefit from preoperative baseline measurements and prospective tracking to establish true changes in health status attributable to hernia repair, with multivariable analysis adjusting for factors such as age and sex.

### 4.4. Limitations

There are several limitations of this study that should be acknowledged. The cross-sectional design lacks prospective baseline PROMs, preventing the quantification of actual QOL changes attributable to surgery; the findings instead reflect descriptive subgroup comparisons. Retrospective preoperative pain assessment is subject to recall bias, and long-term outcomes may be impacted by aging, new comorbidities, or life events. Additionally, the survey coincided with the COVID-19 pandemic, which could have impacted responses and willingness to participate. With a 53% response rate, overall recurrence rates and clinical differences between responders and non-responders is not known, introducing potential non-response bias. Long-term studies may also be subject to survivor bias as older participants may be lost over time, leaving a healthier study population. Furthermore, because all procedures were performed or supervised by a single senior surgeon at one center using an operative strategy dictated by hernia complexity (with significantly more open than laparoscopic repairs), findings may have limited generalizability and cannot be used to compare operative techniques. Subgroup comparisons in this study were therefore exploratory and unadjusted for variables. Larger multicenter studies are needed to better evaluate these associations.

Residual confounding exists as hernia size and case complexity were not evaluated individually. Furthermore, the absence of defined minimal clinically important difference (MCID) thresholds for the used PROMs, combined with the time-consuming nature of administration and a lack of timing consensus, makes interpreting the practical clinical significance of score changes challenging.

## 5. Conclusions

This study shows that patients with high-risk hernias (Grade 3/4) and retrospectively recalled preoperative pain generally report poorer long-term QOL outcomes compared with patients with lower-risk hernias and minimal or no preoperative pain. Nevertheless, favorable long-term functional status and patient satisfaction were observed in many patients in the cohort 6–11 years after ventral primary or incisional hernia repair. These findings contribute to the understanding of long-term patient-reported outcomes and may help inform preoperative counselling and patient expectation setting.

## Figures and Tables

**Table 1:** Comparison of median (range) SF-36 scores.

SF-36 Domain (*n* = 85)	Total Cohort (*n* = 85)	Grade 1 or 2 (*n* = 71)	Grade 3 or 4 (*n* = 16)	*p*-Value	No Pain (*n* = 39)	Pain (*n* = 46)	*p*-Value
Physical Function	10 (0–100)	10 (0–100)	42.5 (0–70)	0.003 *	10 (0–100)	10 (0–80)	0.744
Role limitations due to physical health (*n* = 84)	0 (0–100)	0 (0–100)	0 (0–100)	0.175	0 (0–100)	0 (0–100)	0.546
Role limitations due to emotional problems (*n* = 84)	0 (0–100)	0 (0–100)	0 (0–100)	0.798	0 (0–100)	0 (0–100)	0.943
Energy/fatigue	60 (15–90)	60 (15–90)	52.5 (25–80)	0.096	60 (15–90)	57.5 (25–75)	0.089
Emotional well-being	72 (32–88)	72 (40–88)	72 (32–88)	0.506	76 (52–88)	72 (32–88)	0.201
Social functioning	100 (25–100)	100 (37.5–100)	68.8 (25–100)	0.010 *	100 (25–100)	87.5 (37.5–100)	0.004 *
Pain	75 (25–100)	75 (25–100)	62.5 (25–87.5)	0.013 *	75 (25–100)	68.75 (25–100)	0.196
General health	65 (15–100)	70 (15–100)	47.5 (15–95)	0.007 *	70 (15–100)	65 (15–100)	0.298

* *p* < 0.05.

**Table 2:** Comparison of median (range) patient-reported EuraHS-QOL scores.

EuraHS-QOL Score (*n* = 87)	Total Cohort (*n* = 87)	Grade 1 or 2 (*n* = 71)	Grade 3 or 4 (*n* = 16)	*p*-Value	No Pain (*n* = 41)	Pain (*n* = 46)	*p*-Value
Total score	8 (0–51)	4 (0–43)	10.5 (0–51)	0.030 *	4 (0–25)	8.5 (0–51)	0.087
Pain domain	0 (0–11)	0 (0–10)	0 (0–11)	0.995	0 (0–3)	0 (0–11)	0.019 *
Restriction of activities domain	0 (0–20)	0 (0–16)	0 (0–20)	0.999	0 (0–10)	0 (0–20)	0.060
Cosmetic discomfort domain	8 (0–20)	3 (0–20)	10 (0–20)	0.018 *	3 (0–17)	8 (0–20)	0.174

* *p* < 0.05.

**Table 3:** Comparison of median (range) patient-reported CCS scores.

CCS Score (*n* = 87)	Total Cohort (*n* = 87)	Grade 1 or 2 (*n* = 71)	Grade 3 or 4 (*n* = 16)	*p*-Value	No Pain (*n* = 41)	Pain (*n* = 46)	*p*-Value
Total score (out of 115)	0 (0–62)	0 (0–62)	0.5 (0–19)	0.286	0 (0–24)	0 (0–62)	0.631
Sensation scale (out of 40)	0 (0–2.4)	0 (0–2.25)	0 (0–2.38)	0.585	0 (0–0.8)	0 (0–2.4)	0.222
Pain scale (out of 40)	0 (0–3)	0 (0–3)	0 (0–0.25)	0.635	0 (0–1)	0 (0–3)	0.123
Movement scale (out of 35)	0 (0–2.9)	0 (0–2.86)	0 (0–0.43)	0.626	0 (0–1.6)	0 (0–2.9)	0.739

## Data Availability

The datasets generated from patient surveys are not publicly available. However, de-identified data may be made available by the authors upon reasonable request.

## Supplementary Materials

The following supporting information can be downloaded at https://www.mdpi.com/article/10.3390/jcm15207970/s1, Table S1: Demographic characteristics and operative details of patients; Table S2: Patient-reported outcome response percentages on VHPQ; Figure S1: Comparison of pre- and postoperative pain severity in the VHPQ among ventral hernia patients (*n* = 87) in the long-term follow-up study; Figure S2: Mean values of patient-reported mesh sensation, pain, and movement limitation on CCS; Figure S3: Patient Participation Information Sheet; Figure S4: Postal Questionnaire with Consent Form.

## Abbreviations

The following abbreviations are used in this manuscript:

QOL	Quality-of-life
SSI	Surgical site infection
PROMs	Patient-reported outcome measures
SF-36	36-Item Short-Form Survey
VHPQ	Ventral Hernia Pain Questionnaire
EuraHS-QOL	European Hernia Society Quality of Life
CCS	Carolinas Comfort Scale
VHWG	Ventral Hernia Working Group
MCID	Minimal clinically important difference
