# Supplementary material for: Long-Term Patient-Reported Quality-of-Life Outcomes After Ventral Primary or Incisional Hernia Repair
Source: J Clin Med. 2026 Oct 15;15(20):7970. doi: 10.3390/jcm15207970 (PMC13705729; doi:10.3390/jcm15207970)
Supplement: Supplementary file 1 [file jcm-15-07970-s001.zip › jcm-4569131-supplementary.pdf]

Supplementary Materials

Table S1. Demographic characteristics and operative details of patients

	Total cohort (n=87)	Grade 1 or 2 (n=71)	Grade 3 or 4 (n=16)	P-value	No pain (n=41)	Pain (n=46)	P-value
**Demographics**							
**Median age (range)**	74 (40-97)	74 (41-91)	81 (40-97)	**0.012***	74 (46-93)	74 (40-97)	0.350
**Gender, n (%)**				**0.019***			0.533
**Males**	50 (57)	45 (63)	5 (31)		25 (61)	25 (54)	
**Females**	37 (43)	26 (37)	11 (69)		16 (39)	21 (46)	
**ECOG, n (%)**				**<0.001***			0.999
**0**	55 (63)	52 (73)	3 (19)		26 (63)	29 (63)	
**1**	31 (35)	19 (27)	12 (75)		15 (37)	16 (35)	
**2**	1 (2)	0 (0)	1 (6)		0 (0)	1 (2)	
**Pain or No pain, n (%)**				0.765			
**Pain**	46 (53)	37 (52)	9 (56)				
**No pain**	41 (47)	34 (48)	7 (44)				
**Median follow-up in years (range)**	8 (6-11)	8 (6-11)	7 (6-10)	0.255	8 (6-11)	7.5 (6-11)	0.938
**Operative details**							
**Median age at operation (range)**	62 (27-82)	61 (27-81)	68 (29-82)	**0.009***	63 (35-81)	61 (27-82)	0.320
**Median operative time in minutes (range)**	40 (20-210)	40 (20-150)	70 (35-210)	**<0.001***	40 (20-210)	40 (25-150)	0.454
**Anesthetic, n (%)**				0.999			0.471
**Local**	1 (1)	1 (1)	0 (0)		1 (2)	0 (0)	
**General**	86 (99)	70 (99)	16 (100)		40 (98)	46 (100)	
**Surgical technique, n (%)**	75 (86)	63 (89)	12 (75)	0.221	35 (85)	40 (87)	0.830
**Open**	12 (14)	8 (11)	4 (25)		6 (15)	6 (13)	
**Laparoscopic**							
**Mesh type, n (%)**				**0.038***			0.999
**Polypropylene**	71 (82)	61 (86)	10 (62)		33 (81)	38 (83)	
**Gore-Tex dual mesh**	10 (11)	7 (10)	3 (19)		5 (12)	5 (11)	
**Other**	6 (7)	3 (4)	3 (19)		3 (7)	3 (6)	
**Site, n (%)**				**0.012***			0.168
**Umbilical**	25 (29)	23 (32)	2 (12)		15 (37)	10 (22)	
**Periumbilical**	28 (32)	26 (37)	2 (12)		14 (34)	14 (30)	
**Multifocal**	15 (17)	10 (14)	5 (32)		7 (17)	14 (30)	
**Other**	19 (22)	12 (17)	7 (44)		5 (12)	8 (18)	
**Etiology, n (%)**				**<0.001***			**0.049***
**Primary**	52 (60)	50 (70)	2 (12)		29 (71)	23 (50)	
**Incisional**	35 (40)	21 (30)	14 (88)		12 (29)	23 (50)	
**Recurrent hernia, n (%)**				**0.006***			0.743
**Yes**	10 (11)	5 (7)	5 (31)		4 (10)	6 (13)	
**No**	77 (89)	66 (93)	11 (69)		37 (90)	40 (87)	
**Multifocal, n (%)**				0.101			0.969
**Yes**	15 (17)	10 (14)	5 (31)		7 (17)	8 (17)	
**No**	72 (83)	61 (86)	11 (69)		34 (83)	38 (83)	
**Hernia size, n (%)**				**0.026***			**0.004***
**<2 cm**	45 (52)	41 (58)	4 (25)		28 (68)	17 (37)	
**≥2 cm**	42 (48)	30 (42)	12 (75)		13 (32)	29 (63)	
**Day-only case, n (%)**	50 (57)	48 (68)	2 (12)	**<0.001***	23 (56)	27 (59)	0.807
**Postoperative complications, n (%)**	13 (15)	9 (13)	4 (25)	0.247	4 (10)	9 (20)	0.240

* $p < 0.05$

Table S2. Patient-reported outcome response percentages on VHPQ

Postoperative functional impairment	Yes	No	I don't know	Never performed this activity
Difficulties rising from low-sitting chair	6%	91%	1%	2%
Difficulties sitting down for extended periods	7%	93%	0%	0%
Difficulties standing for extended periods	7%	92%	0%	1%
Difficulties climbing stairs	5%	91%	2%	2%
Difficulties driving a car	0%	94%	2%	3%
Difficulties with exercise	9%	79%	0%	11%
**Postoperative outcomes and satisfaction**	**Yes**	**No**	**I don't know**	
Further abdominal surgery performed?	13%	84%	3%	
Postoperative abdominal stiffness or rigidity?	17%	77%	6%	
Satisfied with surgery?	89%	7%	5%	
Repeat surgery (if necessary)?	80%	8%	11%	

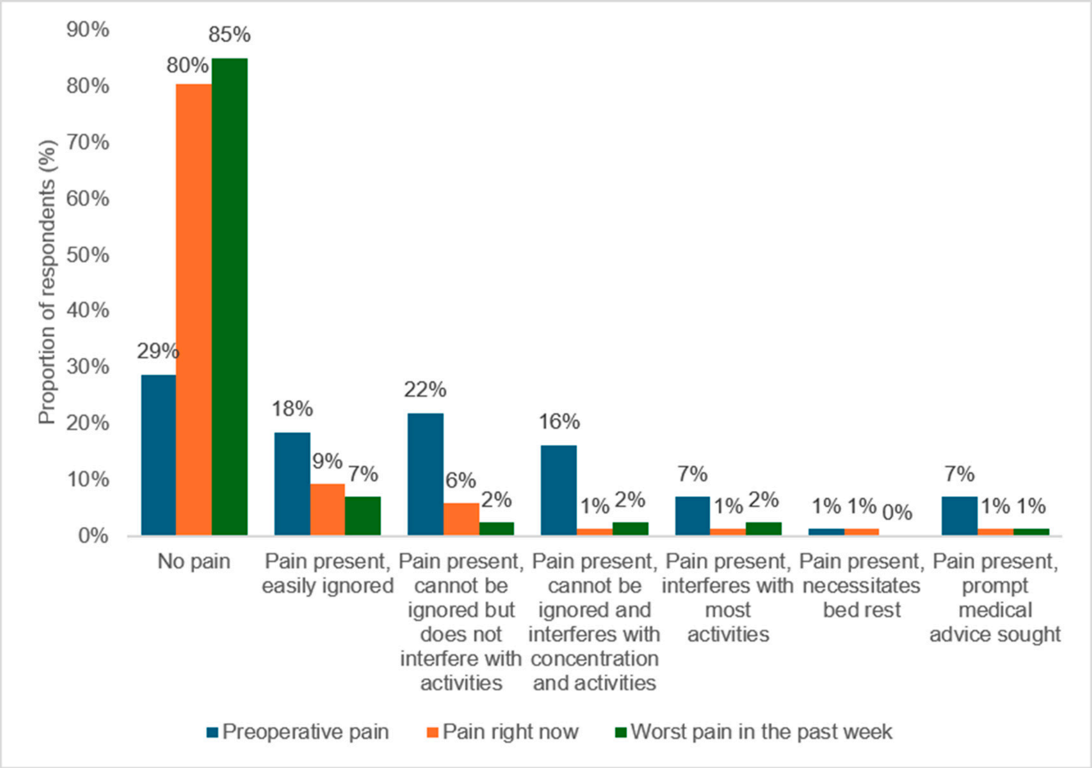

Figure S1. Comparison of pre- and postoperative pain severity in the VHPQ among ventral hernia patients (n = 87) in the long-term follow-up study

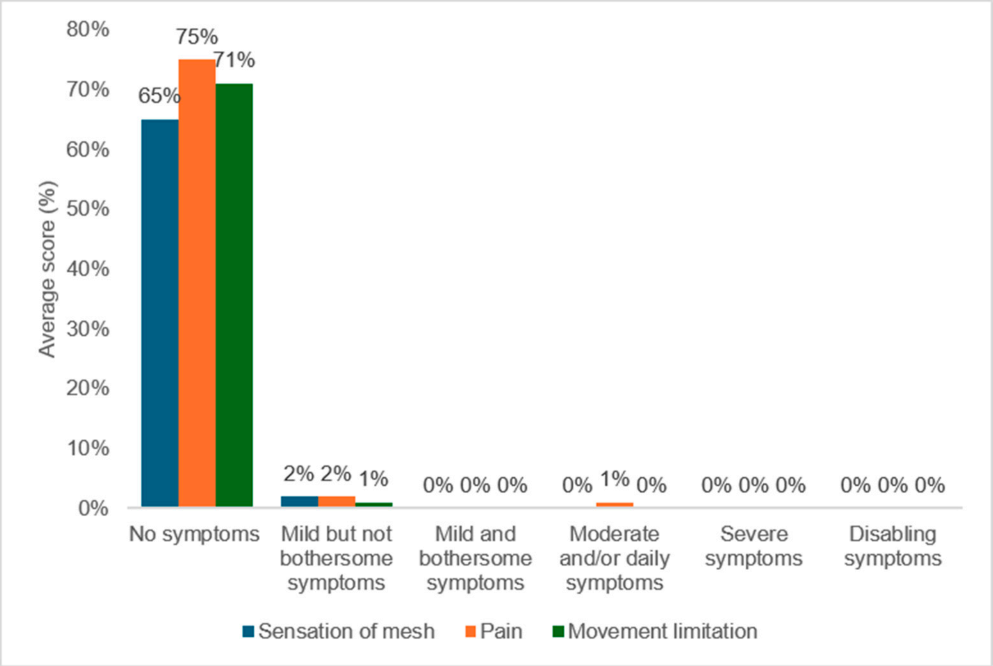

Figure S2. Mean values of patient-reported mesh sensation, pain, and movement limitation on CCS

Health
Northern Sydney
Local Health District

ROYAL NORTH SHORE HOSPITAL

THE UNIVERSITY OF SYDNEY

EVALUATING LONG-TERM PATIENT OUTCOMES IN HERNIA REPAIR

Thank you very much for consideration the invitation to take part in research to improve patient quality of life after hernia surgery. Please take a moment to read the Participant Information Sheet.

If you wish to take part, please complete and return the consent form and survey enclosed using the reply-paid envelope.

Participant Information Sheet

You are invited to participate in a research study into long-term patient outcomes at 5- and 10-years after elective hernia repair surgery.

The study is being conducted by a small team of researchers at the University of Sydney led by Professor Thomas Hugh (Professor of Surgery, Chair of Surgery, Northern Clinical School) and Dr. Joanna Mills (PhD Candidate, Faculty of Medicine and Health, Northern Clinical School).

Before you decide whether you wish to participate in this study, it is important for you to understand why the research is being done and what it will involve. Please take the time to read the following information carefully and discuss it with others if you wish.

1. **'What is the purpose of this study?'**

The purpose is to investigate hernia recurrence, patient-reported quality of life outcomes, and level of satisfaction after elective hernia surgery.

2. **'Why have I been invited to participate in this study?'**

You are eligible to participate in this study because you have previously had an elective hernia operation under the care of Professor Thomas Hugh at either Royal North Shore Hospital or North Shore Private Hospital between January 2009 and December 2014.

3. **'What if I don't want to take part in this study, or if I want to withdraw later?'**

Participation in this study is voluntary. It is completely up to you whether you participate. If you decide not to participate, it will not affect the treatment you receive now or in the future. Whatever your decision, it will not affect your relationship with the investigators, The University of Sydney, Royal North Shore Hospital or North Shore Private Hospital now or in the future.

Being in this study is completely voluntary and you are under no obligation to consent to complete the questionnaire. You can withdraw at any time prior to or after submitting your completed questionnaire. Regardless of whether you participate or not, we will gladly send you a summary of the results if you are interested.

If you wish to withdraw from the study once it has started, you can do so at any time without having to give a reason.

4. **'What does this study involve?'**

If you agree to participate in this study, you will be asked to sign the Participant Consent Form. This study will be conducted over 4 years. Your involvement in the study will involve

Figure S3. Patient Participation Information Sheet

EVALUATING LONG-TERM PATIENT OUTCOMES IN HERNIA REPAIR

CONSENT FORM

1. I, ***[patient name]***
   of ***[patient address]***
   agree to participate as a subject in the study described in the Participant Information Sheet set out above.

2. I acknowledge that I have read the Participant Information Sheet, which explains why I have been selected, the aims of the study and the nature and the possible risks of the investigation, and the statement has been explained to me to my satisfaction.

3. Before signing this consent form, I have been given the opportunity of asking any questions relating to any possible physical and mental harm I might suffer as a result of my participation and I have received satisfactory answers.

4. I understand that I can withdraw from the study at any time without prejudice to my relationship to the investigators or The University of Sydney, Royal North Shore Hospital or North Shore Private Hospital now or in the future.

5. I agree that research data gathered from the results of the study may be published, provided that I cannot be identified.

6. I understand that if I have any questions relating to my participation in this research, I may contact Professor Thomas Hugh and Dr. Joanna Mills on 0407 797 244 or email joanna.mills@sydney.edu.au

7. I acknowledge receipt of a copy of this Consent Form and the Participant Information Sheet.

Complaints may be directed to the Research Office on Level 13, Kolling Building, Royal North Shore Hospital, St Leonards NSW 2065. Phone 02 9926 4590 | email NSLHD-research@health.nsw.gov.au

Signature: **Date:**

Phone Number:
(Required)

Email address:
(Optional)

Health
Northern Sydney
Local Health District

ROYAL NORTH SHORE HOSPITAL

THE UNIVERSITY OF SYDNEY

LONG-TERM PATIENT OUTCOMES IN HERNIA REPAIR

SURVEY

This survey comprises 4 sections:

- Sections 1 to 3 relate to pain or activity limitations resulting from your hernia operation
- Section 4 relates to your views about your general health

SECTION 1
**Instructions:** Please indicate your mesh sensation, pain and mobility levels from 0 (no symptoms) to 5 (disabling symptoms) while performing each of the following activities. Choose N/A for any item that is not applicable to you.<br>**0 = No symptoms**<br>**1 = Mild but not bothersome symptoms**<br>**2 = Mild and bothersome symptoms**<br>**3 = Moderate and/or daily symptoms**<br>**4 = Severe symptoms**<br>**5 = Disabling symptoms**

**1. While laying down, do you have:**							
Sensation of mesh	0	1	2	3	4	5	N/A
Pain	0	1	2	3	4	5	N/A
**2. While bending over, do you have:**							
Sensation of mesh	0	1	2	3	4	5	N/A
Pain	0	1	2	3	4	5	N/A
Movement limitations	0	1	2	3	4	5	N/A
**3. While sitting up, do you have:**							
Sensation of mesh	0	1	2	3	4	5	N/A
Pain	0	1	2	3	4	5	N/A
Movement limitations	0	1	2	3	4	5	N/A
**4. While performing activities of daily living** (i.e. getting out of bed, bathing, getting dressed)**, do you have:**							
Sensation of mesh	0	1	2	3	4	5	N/A
Pain	0	1	2	3	4	5	N/A
Movement limitations	0	1	2	3	4	5	N/A
**5. When coughing or deep breathing, do you have:**							
Sensation of mesh	0	1	2	3	4	5	N/A
Pain	0	1	2	3	4	5	N/A
Movement limitations	0	1	2	3	4	5	N/A
**6. While walking, do you have:**							
Sensation of mesh	0	1	2	3	4	5	N/A
Pain	0	1	2	3	4	5	N/A
Movement limitations	0	1	2	3	4	5	N/A

7. When walking up the stairs, do you have:

Sensation of mesh	0	1	2	3	4	5	N/A
Pain	0	1	2	3	4	5	N/A
Movement limitations	0	1	2	3	4	5	N/A

8. While exercising, do you have:

Sensation of mesh	0	1	2	3	4	5	N/A
Pain	0	1	2	3	4	5	N/A
Movement limitations	0	1	2	3	4	5	N/A

SECTION 2

Instructions: Please rate your level of pain or restriction from 0 (no pain, no restriction) to 10 (worst pain imaginable, completely restricted). Choose N/A for any item that is not applicable to you.

0 = No pain / No restriction
1 - 3 = Mild pain / Mild restriction
4 - 6 = Moderate pain / Mild restriction
7 - 9 = Severe pain / Severe restriction
10 = Worst pain imaginable / Completely restricted

1. Pain at the site of the hernia repair

**Pain in rest** (lying down)	0	1	2	3	4	5	6	7	8	9	10
**Pain during activities** (walking, biking, sports)	0	1	2	3	4	5	6	7	8	9	10
**Pain felt during the last week**	0	1	2	3	4	5	6	7	8	9	10

2. Restrictions of activities because of pain or discomfort at the site of the hernia repair

**Restriction from daily activities** (inside the house)	0	1	2	3	4	5	6	7	8	9	10	N/A
**Restriction outside the house** (walking, biking, driving)	0	1	2	3	4	5	6	7	8	9	10	N/A
**Restriction during sports**	0	1	2	3	4	5	6	7	8	9	10	N/A
**Restriction during heavy labour**	0	1	2	3	4	5	6	7	8	9	10	N/A

Instructions: Please rate your level of cosmetic discomfort out of 10 ranging from 0 (very beautiful) to 10 (extremely ugly).

0 = Very beautiful
1 - 3 = Beautiful
4 - 6 = Neutral
7 - 9 = Ugly
10 = Extremely ugly

3. Cosmetic discomfort

**Shape of your abdomen**	0	1	2	3	4	5	6	7	8	9	10
**Site of the hernia and the scar**	0	1	2	3	4	5	6	7	8	9	10

Health
Northern Sydney
Local Health District

ROYAL NORTH SHORE HOSPITAL

THE UNIVERSITY OF SYDNEY

SECTION 3

Instructions: Please indicate your pain level using each drop-down list from 0 (no pain) to 6 (pain so severe that you were forced to seek medical attention).
0 = No pain
1 = Pain that could easily be ignored
2 = Pain that could not be ignored but did not influence daily activities
3 = Pain that could not be ignored, which affected concentration and performance of daily activities
4 = Pain that inhibited most daily activities
5 = Pain that required rest or bed rest
6 = Pain so severe that you were forced to seek medical attention

1. Describe the pain you experienced from your hernia prior to surgery.
0 1 2 3 4 5 6

2. Describe the abdominal pain you experience right now following surgery.
0 1 2 3 4 5 6

3. Describe your abdominal pain when most intense during this last week.
0 1 2 3 4 5 6

4. If you no longer have pain in the operated area, try to recall when your abdominal pain stopped.
- ☐ I still have abdominal pain
- ☐ Pain in the operated area stopped within 1 month following surgery
- ☐ Pain in the operated area stopped within 3 months following surgery
- ☐ Pain in the operated area stopped within 6 months following surgery
- ☐ Pain in the operated area stopped within 1 year following surgery
- ☐ Pain in the operated area stopped within 2 years following surgery
- ☐ Pain in the operated area stopped recently

5. How often have you felt abdominal pain in the operated area in the past week?
- ☐ No pain
- ☐ A few times during the last week
- ☐ Several times during the last week
- ☐ Every day
- ☐ Every day and night
- ☐ Constant pain during the last week (day and night)

6. How long have the episodes of pain lasted in the past week?
- ☐ No pain
- ☐ A few minutes
- ☐ Several minutes
- ☐ Most of the day
- ☐ All day
- ☐ Constant pain during the last week (day and night)

7. **Do you find it difficult to rise from a low-sitting chair as a result of your abdominal pain?**
   - ☐ No
   - ☐ Yes
   - ☐ Not sure
   - ☐ Never perform this activity

8. **Do you find it difficult to sit for an extended period (over 30 minutes) as a result of your abdominal pain?**
   - ☐ No
   - ☐ Yes
   - ☐ Not sure
   - ☐ Never perform this activity

9. **Do you find it difficult to stand for an extended period (over 30 minutes) as a result of your abdominal pain?**
   - ☐ No
   - ☐ Yes
   - ☐ Not sure
   - ☐ Never perform this activity

10. **Do you find it difficult to climb stairs as a result of your abdominal pain?**
    - ☐ No
    - ☐ Yes
    - ☐ Not sure
    - ☐ Never perform this activity

11. **Do you find it difficult to drive a car as a result of your abdominal pain?**
    - ☐ No
    - ☐ Yes
    - ☐ Not sure
    - ☐ Never perform this activity

12. **Has abdominal pain limited your ability to perform sports activities?**
    - ☐ No
    - ☐ Yes
    - ☐ Not sure
    - ☐ Never perform this activity

13. **Have you taken any pain medication during the last week for abdominal pain?**
    - ☐ No
    - ☐ Yes
    - ☐ I don't know

If you answered "Yes," which medication?

14. To what extent has abdominal pain limited your working capability in the last 2 months?

- ☐ I have not needed any sick-leave as a result of abdominal pain
- ☐ Abdominal pain has caused 1-7 days of sick-leave during the last 2 months
- ☐ Abdominal pain has caused 1-4 weeks of sick-leave during the last 2 months
- ☐ Abdominal pain has caused constant sick-leave during the last 2 months
- ☐ Abdominal pain has caused me to seek disability income
- ☐ I am unemployed or retired

15. Have you had another hernia operation at the same site or anywhere else in your abdomen (including prostate surgery) after the hernia operation that Professor Hugh performed?

- ☐ No
- ☐ Yes
- ☐ I don't know

If you answered "Yes," what kind of operation?

- ☐ My hernia recurred and was operated on by Professor Hugh
- ☐ My hernia recurred and was operated on by someone else
- ☐ A new groin hernia
- ☐ A new ventral/incisional hernia
- ☐ An abdominal operation
- ☐ A prostatectomy
- ☐ I don't know

16. Do you feel any abdominal stiffness or rigidity after surgery?

- ☐ No
- ☐ Yes
- ☐ I don't know

17. Are you satisfied with the hernia operation that Professor Hugh performed?

- ☐ No
- ☐ Yes
- ☐ I don't know

18. Would you repeat the operation if necessary?

- ☐ No
- ☐ Yes
- ☐ I don't know

19. How would you describe your work?

- ☐ Heavy physical work
- ☐ Light physical work
- ☐ Office work
- ☐ I am unemployed or retired

SECTION 4

1. In general, would you say your health is:

- ☐ Excellent
- ☐ Very good
- ☐ Good
- ☐ Fair
- ☐ Poor

2. Compared to one year ago, how would you rate your health in general now?

- ☐ Much better now than one year ago
- ☐ Somewhat better now than one year ago
- ☐ About the same
- ☐ Somewhat worse now than one year ago
- ☐ Much worse now than one year ago

The following items are about activities you might do during a typical day. Does your health now limit you in these activities? If so, how much?

3. Vigorous activities, such as running, lifting heavy objects, participating in strenuous sports

- ☐ No, Not limited at all
- ☐ Yes, Limited a lot
- ☐ Yes, Limited a little

4. Moderate activities, such as moving a table, pushing a vacuum cleaner, bowling, or playing golf

- ☐ No, Not limited at all
- ☐ Yes, Limited a lot
- ☐ Yes, Limited a little

5. Lifting or carrying groceries

- ☐ No, Not limited at all
- ☐ Yes, Limited a lot
- ☐ Yes, Limited a little

6. Climbing several flights of stairs

- ☐ No, Not limited at all
- ☐ Yes, Limited a lot
- ☐ Yes, Limited a little

7. Climbing one flight of stairs

- ☐ No, Not limited at all
- ☐ Yes, Limited a lot
- ☐ Yes, Limited a little

8. Bending, kneeling, or stooping
- ☐ No, Not limited at all
- ☐ Yes, Limited a lot
- ☐ Yes, Limited a little

9. Walking more than a mile (1.6km)
- ☐ No, Not limited at all
- ☐ Yes, Limited a lot
- ☐ Yes, Limited a little

10. Walking several blocks
- ☐ No, Not limited at all
- ☐ Yes, Limited a lot
- ☐ Yes, Limited a little

11. Walking one block
- ☐ No, Not limited at all
- ☐ Yes, Limited a lot
- ☐ Yes, Limited a little

12. Bathing or dressing yourself
- ☐ No, Not limited at all
- ☐ Yes, Limited a lot
- ☐ Yes, Limited a little

During the past 4 weeks, have you had any of the following problems with your work or other regular daily activities as a result of your physical health?

13. Cut down on the amount of time you spent on work or other activities
- ☐ No
- ☐ Yes
- ☐ I don't know

14. Accomplished less than you would like
- ☐ No
- ☐ Yes
- ☐ I don't know

15. Were limited in the kind of work or other activities
- ☐ No
- ☐ Yes
- ☐ I don't know

16. Had difficulty performing the work or other activities (e.g. it took extra effort)
- ☐ No
- ☐ Yes
- ☐ I don't know

During the past 4 weeks, have you had any of the following problems with your work or other regular daily activities as a result of any emotional problems (such as feeling depressed or anxious)?

17. Cut down the amount of time you spent on work or other activities

- ☐ No
- ☐ Yes
- ☐ I don't know

18. Accomplished less than you would like

- ☐ No
- ☐ Yes
- ☐ I don't know

19. Didn't do work or other activities as carefully as usual

- ☐ No
- ☐ Yes
- ☐ I don't know

20. During the past 4 weeks, to what extent has your physical health or emotional problems interfered with your normal social activities with family, friends, neighbours, or groups?

- ☐ Not at all
- ☐ Slightly
- ☐ Moderately
- ☐ Quite a bit
- ☐ Extremely

21. How much bodily pain have you had during the past 4 weeks?

- ☐ None
- ☐ Very mild
- ☐ Mild
- ☐ Moderate
- ☐ Severe
- ☐ Very severe

22. During the past 4 weeks, how much did pain interfere with your normal work (including both work outside the home and housework)?

- ☐ Not at all
- ☐ Slightly
- ☐ Moderately
- ☐ Quite a bit
- ☐ Extremely

These next questions are about how you feel and how things have been with you during the past 4 weeks. For each question, please give the one answer that comes closest to the way you have been feeling.

Instructions: Please indicate how much of the time you have been feeling this way using each drop-down list from 1 (all of the time) to 5 (none of the time).
1 = All of the time
2 = Most of the time
3 = Some of the time
4 = A little of the time
5 = None of the time

How much of the time during the past 4 weeks...

23. Did you feel full of pep (high spirits)?
1 2 3 4 5

24. Have you been a very nervous person?
1 2 3 4 5

25. Have you felt so down in the dumps that nothing could cheer you up?
1 2 3 4 5

26. Have you felt calm and peaceful?
1 2 3 4 5

27. Did you have a lot of energy?
1 2 3 4 5

28. Have you felt downhearted and blue?
1 2 3 4 5

29. Did you feel worn out?
1 2 3 4 5

30. Have you been a happy person?
1 2 3 4 5

31. Did you feel tired?
1 2 3 4 5

32. How much of the time has your physical health or emotional problems interfered with your social activities (like visiting with friends, relatives, etc.)?
1 2 3 4 5

Health
Northern Sydney
Local Health District

ROYAL NORTH SHORE HOSPITAL

THE UNIVERSITY OF SYDNEY

Instructions: Please indicate how true or false the following statements are for you using each drop-down list from 1 (definitely true) to 5 (definitely false).
1 = Definitely true
2 = Mostly true
3 = Don't know
4 = Mostly false
5 = Definitely false

How TRUE or FALSE is each of the following statements for you.

33. I seem to get sick a little easier than other people
1 2 3 4 5

34. I am as healthy as anybody I know
1 2 3 4 5

35. I expect my health to get worse
1 2 3 4 5

36. My health is excellent
1 2 3 4 5

Sydney Hernia Study, ***[patient code]*** *- Page 11 of 11*

Figure S4. Postal Questionnaire with Consent Form
